# Microfluidic-based flexible reflective multicolor display

**DOI:** 10.1038/s41378-018-0018-1

**Published:** 2018-07-16

**Authors:** Kazuhiro Kobayashi, Hiroaki Onoe

**Affiliations:** School of Integrated Design Engineering, Graduate School of Science and Technology, Keio University, Yokohama, Japan

## Abstract

This paper describes a microfluidic-based flexible reflective display constructed using dyed water droplets and air gaps as pixel elements. Our display is composed of a flexible polydimethylsiloxane sheet with a connected pixel-patterned microchannel. Several types of dyed water droplets and air gaps are sequentially introduced to the microchannel through a suction process to display a multicolor image. The displayed image is stable and can be retained without an energy supply. To ensure that images are displayed correctly, the geometric parameters of the dot pixel design and minimum differential pressure necessary to drive the water droplets are evaluated. As a demonstration, we successfully display three-color dot-matrix reflective images and bitmap characters in the microchannel. Our proposed method can be applied to energy-less and color-changeable displays for use in future daily-life accessories, such as bags, shoes, and clothes, and can change the surface color and pattern of these accessories.

## Microfluidics: flexible displays

Sequentially introducing colored water droplets and air pockets into a microfluidic device based on a flexible polymer enables a display with zero power consumption. Kazuhiro Kobayashi and Hiroaki Onoe from Keio University in Japan report a multicolored flexible display based on sequentially pumping colored water, followed by air, through polydimethylsiloxiane-based channels. Single water droplets define individual pixels, which are separated by air gaps; pumping water and air through the device in sequence allows colored patterns to be displayed, which can be controlled by varying the sequence of water and air, as well as water color. Additionally, retaining the pattern does not require the input of energy, while polydimethylsiloxiane makes the device flexible. Such a device could find application in displays on clothes and the skin of robots.

## Introduction

Since ancient times, the use of pictures, letters, and symbols has been crucial for recording and expressing the information necessary to advance human culture and civilization^1–3^. In modern societies, books, newspapers, and signs are used to convey information, with art forms, such as painting, clothing, and accessories, expressing individuality. From the viewpoint of material science, letters and pictures are equivalent to the patterns of dyes (black lead, ink, etc) on the surface of a base material (paper, board, cloth, etc). These patterned dyes are visualized by the reflection of ambient light, and as long as the dyes remain stable on the surface of the base material, it is possible to display images for a long time without a need for energy consumption.

In recent years, a technology that enables the electronic rewriting of letters and pictures by controlling the position or color of the dye has been developed. This technology has now been recognized as a reflective display (or electric paper) with low-energy consumption and eye-friendly features for displaying still images^4^. The key technical point of these reflective displays is that they can continue to show images with almost no energy consumption, enabling their use as substitutes for traditional letters or pictures (patterned dyes) on paper. This characteristic is different from other light-emitting displays, such as liquid crystal displays^5^, organic light-emitting diode displays^6^, thin-film electroluminescent displays^7^, vacuum fluorescent displays, and field emission displays^8^, which can rapidly change their images, such as to play movies, but consume electrical energy to display the color of each light-emitting pixel.

The major principle to construct these types of reflective displays is the use of electric ink^9–12^. Electronic ink can continue to display images without consuming energy by using particle elements for pixels and show colors by controlling the position or characteristics of the particles. Typically, when displaying an image, particles with hemispheres of different colors are rotated by electrophoresis or differently colored particles are moved by dielectrophoresis to change each pixel’s color^9–11^. Energy is required to change the image, but retaining a single image does not use any energy. Thus, this method can be used to give the effect of changeable paper. However, technical limitations mean that only two or three colors can be displayed. Thus, multi-colorization of images is the next challenge for electric-ink-based reflective displays.

To realize colorful reflective displays, one of the emerging technologies is the use of liquid to display elements^13^, for example, in displays using the electrowetting phenomenon^14–16^ and wall-hanging signboards using a magnetic fluid^17^. Particularly, displays using dyed liquid and patterned microfluidic channels have been actively developed^18,19^ with the technical advancements in the field of microfluidics. These systems display images by introducing colored fluid into a microfluidic device. Therefore, a color image can be maintained without consuming energy after introducing the fluid. However, arbitrary pixel images cannot be displayed for each color because the displayed image is defined by the geometry of the microfluidic network. Hence, a novel approach is required to develop a microfluidic-based color reflective display that can display and retain any image without consuming energy.

**Fig. 1:**
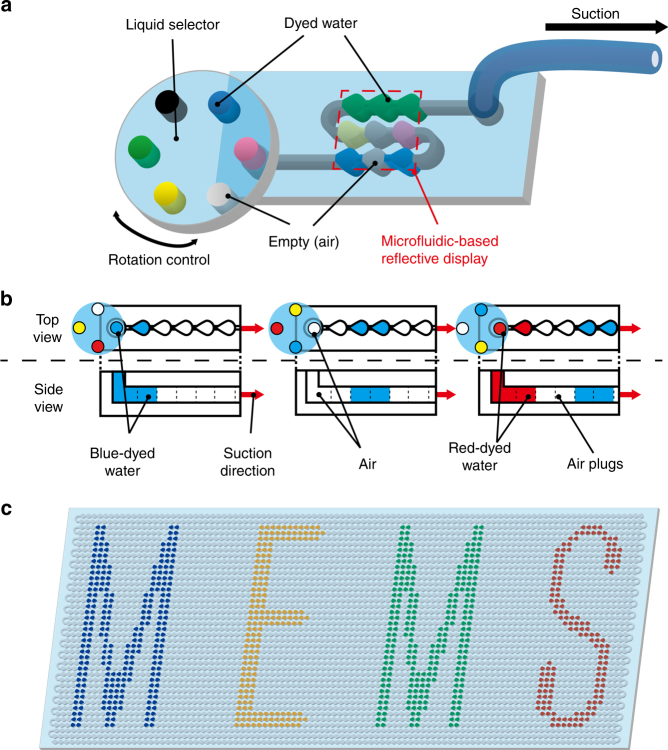
Conceptual illustration of our microfluidic-based reflective display using dyed water as pixel elements. **a** Principle diagram of microfluidic-based reflective display. Several types of dyed water and air, selected by a rotary liquid collector, are sequentially introduced to the microchannel by suction. **b** Working principle for introducing multiple dyed water droplets and air plugs inside the microchannel. Air plugs work as blank pixels and separate each dyed water droplet. **c** Example image created with our device. Multicolored reflective images can be displayed with patterned dyed water droplets and maintained with no energy consumption

In this paper, we present a microfluidic-based water-droplet-train flexible reflective display that is able to show and retain colored images with no energy consumption. This system uses a single-layered dot-connected microfluidic device in which dyed water is used for pixel elements and transparent air plugs separate each water pixel. Several types of dyed water and air are sequentially introduced to the microchannel to display color images. Each dyed water droplet works as a colored pixel to display images. For selective dyed water injection, we use a rotary liquid selector and suction-based negative pressure to drive the droplets (Fig. 1). This system is constructed using flexible polydimethylsiloxane (PDMS) to create a display that is as bendable as paper. In the remainder of this paper, we first evaluate the relationship between the pixel size and shape and the accuracy of the display to investigate the optimal dot shape. Next, we evaluate the pressure response of water movement when using this optimal dot shape. Finally, we demonstrate the images and bending that can be achieved by our display.

## Materials and methods

### Chemicals

Silicon wafers (4 in) were purchased from Matsuzaki Seisakusyo Co., Ltd. (Fukui, Japan), Acetone, isopropyl alcohol (IPA), and ethanol were purchased from Wako Co. Negative photoresists (SU-8 3050 and SU-8 3025), and SU-8 developer solution was purchased from Nippon Kayaku Co. (Tokyo, Japan). PDMS prepolymer and a curing agent kit (SILPOT 184) were purchased from TORAY (Tokyo, Japan). Water soluble color dyes (red: 14% new coccine (C_20_H_11_N_2_Na_3_O_10_S_3_) and 86% dextrin; blue: 8% Brilliant Blue FCF (C_37_H_34_N_2_Na_2_O_9_S_3_) and 92% dextrin); and yellow: 14% tartrazine (C_16_H_9_N_4_Na_3_O_9_S_2_) and 86% dextrin) were purchased from Kyoritsu-foods Co. (Tokyo, Japan). The purchased dye powders were dissolved in deionized water at 0.5% (w/w) for our experiments. Deionized water was obtained from the Direct-Q UV 3 (Merck Millipore, MA, USA) 224 purification system. Parylene C was purchased from Specialty Coating Systems (IN, USA).

### Device fabrication

The microfluidic channel device was made of PDMS (the detailed fabrication process is shown in Figs. S1–1). Briefly, molds for the microfluidic channels were fabricated on a silicon wafer using standard photolithography techniques. A silicon wafer was coated with negative photoresist (SU-8 3050) using a spin coater and photolithographically patterned. The PDMS prepolymer and curing agent were mixed at a 10:1 ratio and cured on the patterned SU-8 mold, thoroughly degassed in vacuum, and cured to form pixel-patterned microchannels. Simultaneously, the mixture was cast onto another silicon wafer with spin coating to form a PDMS sheet (~20 μm). After curing, both the PDMS microchannel and sheet were peeled and bonded after oxygen plasma treatment. After bonding, we punched the PDMS microchannels to make holes for an inlet and an outlet for tubing. Finally, we deposited an approximately 5 nm-thick Parylene C film inside the microchannel using a parylene vapor deposition apparatus (PDS-2010, Japan Parylene Co., Tokyo, Japan) to prevent air leakage into the microchannel because Parylene C has high gas barrier characteristics.

The liquid selector was made of PDMS. The PDMS prepolymer and curing agent were mixed at a 10:1 ratio and cured on a sterols Petri dish, thoroughly degassed in vacuum, and cured. After curing, we punched six holes at 60° intervals 2 cm from the center of the liquid selector. Finally, we deposited Parylene C on the surface.

### Computer-controlled valve system

**Fig. 2:**
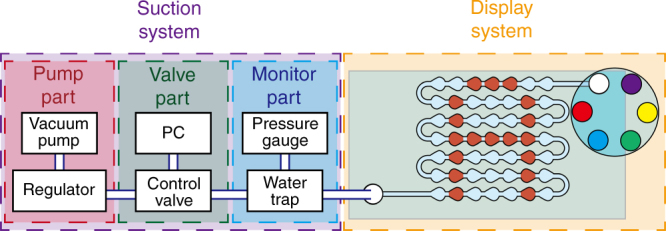
**Conceptual diagram of computer-controlled valve system for positioning droplets in a microchannel to display images**

To control the pressure inside the microfluidic device, we used a computer-controlled valve system (Fig. 2). This system was composed of a computer-controlled solenoid valve, vacuum pump, and air regulator that was able to switch the output pressure to either atmospheric pressure or gauge pressure over the range −3.5–0 kPa. The switch control was programed by MATLAB, and the gauge pressure was controlled by the regulator (Fig. 2).

### Evaluation of volume loss of water dot pixels

**Fig. 3:**
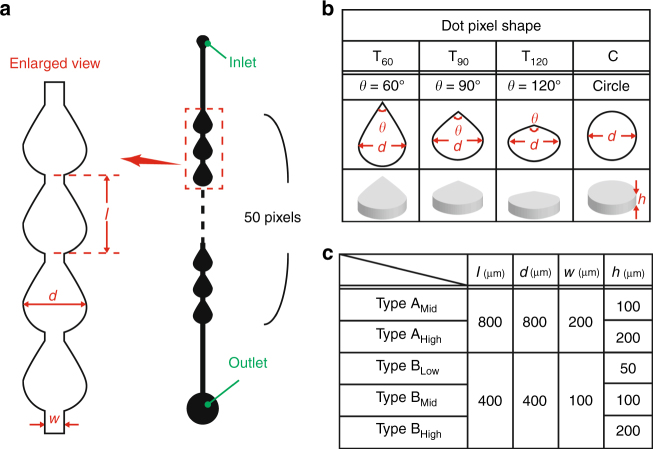
Geometric parameters for dot pixels and microchannels for evaluating the loss of dyed water droplets. **a** Enlarged view of the microchannel. **b** Four types of dot pixel shapes. **c** Five types of microchannel (two-pixel diameters and three microchannel heights were examined)

Several types of linear microchannels with various dot shapes and sizes (Fig. 3) were tested to evaluate the volume loss of water droplets in the microchannels. Continuous dots (3, 5, or 7 dots) of dyed water sandwiched between air plugs were introduced into the microchannel through a suction process by applying negative pressure to the outlet port. Images of the introduced continuous water dots in the initial state were then captured from above by a microscope (VH-5500, KEYENCE, Osaka, Japan). Negative pressure was again applied to make the continuous dyed water dots advance by seven dot pixels in the microchannel, and additional microscopic images were captured. These processes were repeated 15 times, corresponding to a total pixel shift of 105 dots from the initial state. The volume loss of the continuous water dot pixels was evaluated by calculating the remaining volume of water at each position as follows. The total area of the water dot pixels was measured by image analysis using ImageJ software (NIH), and the height of the microchannel was estimated by measuring the height of the mold with a laser profile meter (VK-X100, KEYENCE). The volume of the continuous dots was obtained by multiplying the area by the channel height.

To examine the relationship between the volume loss of water droplets and the microchannel geometries, four 50-pixel straight microchannels with different pixel shapes (teardrop shapes with inflow tapered angles of *θ* = 60°, 90°, 120° (T_60_, T_90_, T_120_) and a circle (C)) were designed. This design enabled us to investigate the influence of the inflow angle on the pixels (Fig. 3). For each type of microchannel, we prepared two-pixel diameters (Type A: *d* = 800 µm and Type B: *d* = 400 µm) and three microchannel heights (*h* = 50 µm (low), 100 µm (mid), and 200 µm (high)). Note that we were unable to fabricate a microchannel with *d* = 800 µm and *h* = 50 µm (Type A_Low_) because some parts of the microchannel collapsed. We define the pixel loss of a dyed water droplet, *W*_loss_(*n*) as follows:

$$W_{{\mathrm{loss}}\,}\left( n \right) = \frac{{V\left( 0 \right) - V\left( {n\prime } \right)}}{{V_{{\mathrm{pixel}}} \cdot n\prime }} \cdot n,$$

where *n* (independent variable) and *n′* (experimental constant) are the numbers of pixels the water droplet advances in a microchannel (for initial position, *n* = 0), *V*(*n*) is the volume of the water droplet after advancing along the microchannel *n* pixels (*V*(0) represents the initial volume of the water droplet), and *V*_pixel_ is the volume of one pixel in the microchannel. The value of [*V*(0) − *V*(*n′*)]/*V*_pixel_∙*n′* was experimentally obtained (*n′* = 7) and averaged over *N* = 15 measurements for each microchannel.

### Pressure measurement for advancing dyed water dots in microchannels

A Type A_high_ channel was used for the pressure measurement. Continuous water dots (1–10 dots) sandwiched between air plugs were introduced into the microchannel using negative pressure and settled by releasing the negative pressure. Then, we gradually raised the negative gauge pressure again, stopped raising the pressure when the continuous water dots started to move in the microchannel, and recorded the pressure required to advance the dyed water dots in the microchannel. In addition, we measured the pressure required to move continuous water dots (2–10 dots) that contained one or two internal air dots to examine the effect of the number of water–air boundaries.

### Demonstration of multicolor reflective display

Red (0.5% (w/w)), yellow (0.5% (w/w)), and blue (0.5% (w/w)) dyed water droplets and air (blank) were prepared in reservoirs of the liquid selector. By controlling the negative pressure generated by the valve system, we sequentially introduced dyed water and air alternately to generate multicolor dyed water droplet patterns in linear microchannels for one-dimensional displays or meandering (zigzag) microchannels for two-dimensional displays. To test the display performance, we introduced dyed water and air alternately and formed images in the matrix channels through the multicolor dot matrix. We changed the dye color at each alternation. To evaluate the color display performance, the resulting images were captured by a digital camera (GXR, Ricoh Imaging Co., Tokyo, Japan) or a digital microscope (VH-5500, KEYENCE).

## Results

### Fabricated microfluidic display

**Fig. 4:**
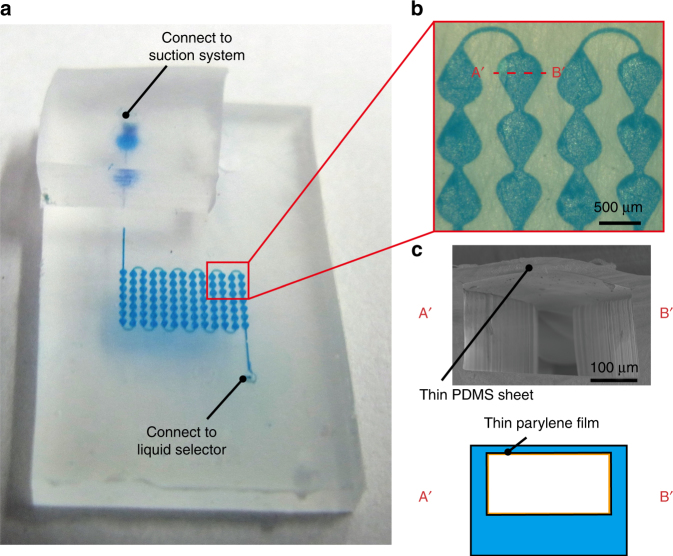
Fabricated PDMS microchannel. **a** Meandering (zigzag) microchannel with a 7 × 13 pixels (25 dpi) display. Inlet and outlet ports were connected to the liquid selector and suction system, respectively. **b** Microscopic image of the microchannel with teardrop-shaped pixels. Note that the white dots in each pixel were caused by reflections of illuminated light on the device surface. **c** Cross-sectional view of the microchannel. The top of the microchannel is a thin PDMS sheet with a parylene film deposited inside the microchannel to prevent air leakage

The microchannels for the multicolor reflective microfluidic display were made of flexible PDMS, which is highly transparent under visible light wavelengths. Thus, our display is able to show patterned dyed water droplets inside the channel, which can easily be recognized as a display image, and the display can bend like paper. Using soft lithography and bonding techniques, PDMS-PDMS microchannels containing pixel patterns (typically 400–800 μm in diameter and 50–200 μm in height) were connected with linear channels (100–200 μm in width) (Fig. 4a). As PDMS is permeable to air^20^, a thin parylene layer (500 nm thick) was deposited inside the microchannel (Fig. 4c) to prevent the leakage of air and water evaporation^21^.

### Evaluation of the volume loss of water dot pixels

To show an image in our display correctly, it is important to maintain the volume of dyed water droplets while they advance through the microchannel. If some of the water remains on the microchannel wall, the water droplet volume will decrease and the displayed image will be disturbed. Such water residues in microchannels occur because of the physical characteristics and geometric parameters of both the microchannel and fluid, such as the shape of the dot pixels, height, and width of the microchannel, and contact angle between the fluid and microchannel wall.

**Fig. 5:**
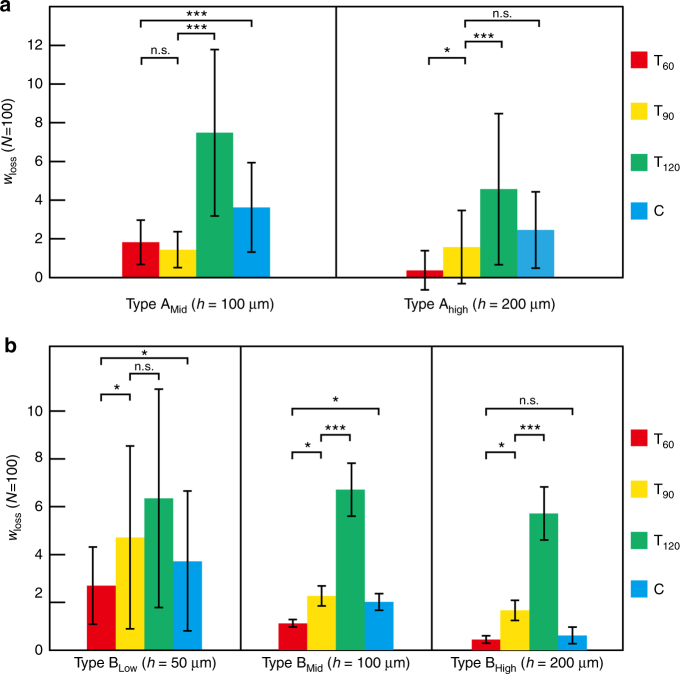
Relationship between microchannel geometries and *W*_loss_ at *n* = 100. Error bars indicate standard deviation (*N* = 15). (Note: * means *P* < 0.05, *** means *P* < 0.005). **a** Type A channel. **b** Type B channel

Figure 5 shows the relationship between the microchannel geometry and *W*_loss_(100), which reflects the water loss when the water droplet advances 100 pixels. For the Type A microchannel (*d* = 400 µm), the values of *W*_loss_(100) for T_60_ and T_90_ dot pixels were much lower than those of other shapes (T_120_ and C), regardless of the microchannel height. This result indicates that dot pixels with sharp-tapered angles (*θ* < 90°) result in a lower *W*_loss_ than dot pixels with larger-tapered angles or circular shapes.

As for Type B microchannels (*d* = 200 µm), *W*_loss_(100) decreased as the inflow taper angle became smaller, which is similar to the case for Type A microchannels (*d* = 400 µm). However, compared to Type A microchannels, the standard deviation of *W*_loss_(100) drastically decreased for Type B_mid_ and Type B_high_ microchannels, which have smaller pixel diameters (*d* = 200 µm) and larger heights (*h* = 100 µm or 200 µm). In particular, *W*_loss_(100) for pixel shapes with the sharpest taper angle (T_60_) was significantly lower and exhibited little variation (1.12 ± 0.29, 0.45 ± 0.16 for Type B_mid_ and Type B_high_, respectively) compared with other pixels with larger taper angles (T_90_ and T_120_) (*p < 0.05, ***p < 0.005, *N* = 15). The circular pixels (C) also gave low *W*_loss_(100) values with large channel heights (0.62 ± 0.35 for Type B_high_). These results indicate that water can be stably transferred inside the microchannel when the inflow taper angle is small and the microchannel is relatively deep.

In summary, we found that T_60_ pixels with Type B_high_ microchannel gave the best performance (i.e., the lowest *W*_loss_(100)) among our candidates. Thus, we used this design for the experiments and demonstrations described below.

### Minimum differential pressure required to drive water droplets in microchannels

**Fig. 6:**
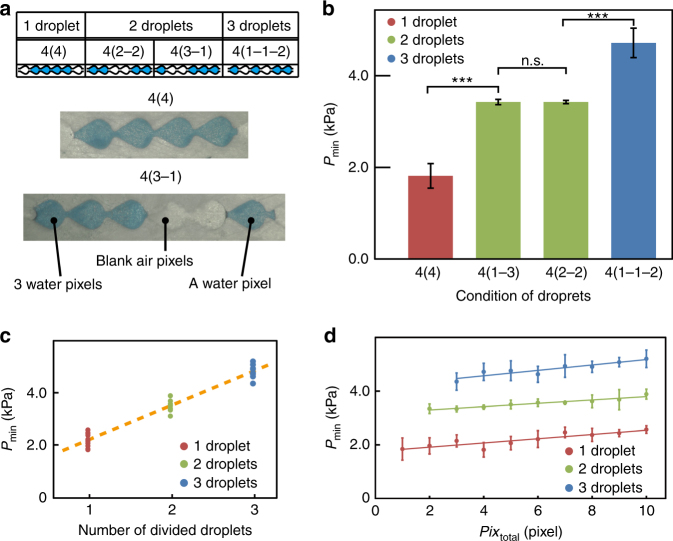
Minimum differential pressure for driving dyed water droplets. **a** Definition of the condition of water droplets. **b** Relationship between minimum differential pressure, *P*_min_, and the condition of four-pixel water droplets. **c** Relationship between *P*_min_ and total number of pixels, *P*_ix_ total. **d** Relationship between *P*_min_ and number of droplets, *m*

To control the position of dyed water droplets inside the microchannel, we evaluated the minimum differential pressure for driving the dyed water droplets, *P*_min_. As the volume of the dyed water droplets or the number of droplets in the microchannels increased, the pressure required to advance the droplets increased. To describe the condition of the introduced dyed water droplets, we define *Pix*_total_(*Pix*_1_-*Pix*_2_-…-*Pix*_m_) to represent the total number of pixels occupied by the introduced dyed water droplets, where *Pix*_i_ is the number of pixels covered by each water droplet (*i* = 1, 2,..., *m*) when the introduced dyed water is divided into *m* droplets. For example, as shown in Fig. 6a, a four-pixel continuous droplet is expressed as 4(4), but four-pixel droplets composed of three-pixel continuous droplets and a single-pixel droplet with air blank pixels are expressed as 4(3-1). Using the four-pixel conditions of 4(4), 4(1-3), 4(2-2), and 4(1-1-2), we examined the minimum differential pressure *P*_min_. Figure 6b shows that there were significant differences between three groups: (i) 4(4), (ii) 4(1-3) and 4(2-2), and (iii) 4(1-1-2). No significant differences were observed between the two conditions in group (ii). Note that there was no significant difference between the volume loss of dyed water pixels and number of pixels in continuous droplets (Figure S4). This result indicates that *P*_min_ depends not only on the number of divided droplets *m*, but also on the total pixel number (volume) of introduced dyed water droplets, *Pix*_total_, and the volume balance between the divided droplets, *Pix*_1_ and *Pix*_2_.

To confirm the relationship between *P*_min_, *m*, and *Pix*_total,_ we performed experiments using pixel conditions with *Pix*_total_ from 1–10 and *m* = 1, 2, and 3 (Fig. 6c, d). As *m* increased, *P*_min_ increased linearly by 1.25 kPa/(number of divided pixels) (Fig. 6c). This result indicates that the number of interfaces between dyed water/air determines the resistance against advancing dyed water droplets in the microchannels, resulting in an increase in the required pressure difference, *P*_min_. The pressure was less sensitive to increases in *Pix*_total_, but increased slightly (~0.1 kPa/pixel for all three droplet conditions) (Fig. 6d). This slight increase might be caused by a difference in pressure loss and flow friction in the microchannel.

### Control of multicolored droplets in microchannels

**Fig. 7:**
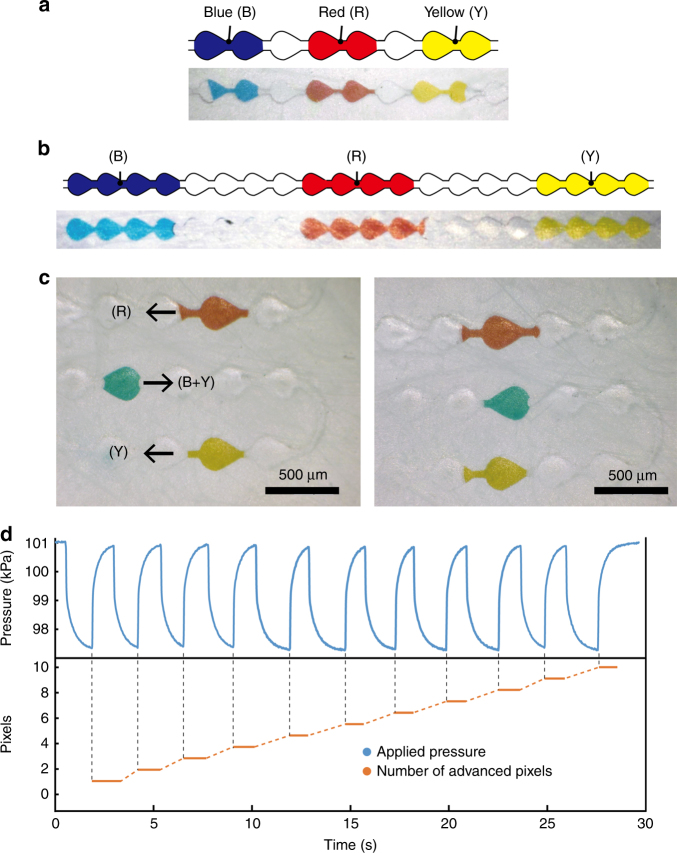
Control of droplets in the microchannel. **a** Two-pixel droplets separated by single-pixel air gaps. **b** Four-pixel droplets separated by four-pixel air gaps. **c** Single-pixel position control of droplets. **d** Relationship between applied negative pressure and pixel position of a droplet in the microchannel

Using the microchannel design and pressure control described above, we first tested the color switching performance of our microfluidic-based display. We alternately introduced dyed water (blue, red, and yellow) and blank air plugs into a linear microchannel. Differently colored two-pixel droplets separated by single air blank pixels (Fig. 7a) and four-pixel droplets gapped by four air blank pixels (Fig. 7b) were successfully displayed, showing that this display enables color switching using two-color pixels with a one-pixel blank.

In addition to color switching, controlling the position of the droplets at the single-pixel level is an important function for displaying an image. We designed meandering multi-line microchannels as a test microfluidic display with 7 × 5 pixels (50 dpi) and evaluated the position control of the introduced dyed water droplets. Three single-pixel droplets were properly aligned by advancing a single pixel using the applied negative pressure (Fig. 7c). The relationship between the droplet positions and timing of the applied negative pressure clearly indicated that the position of droplets could be controlled at the single-pixel level (Fig. 7d and Supporting Movie 1). Furthermore, there was no difference in the behavior of each droplet in the microchannel because water droplets dyed with different pigments (R, Y, and B × Y) had almost the same physical properties (e.g., viscosity (~1.0 mPas) and surface tension (~72 mN/m)). This feature enabled us to use a wide range of color variations in our display by mixing droplets with primary colors.

### Display demonstrations

**Fig. 8:**
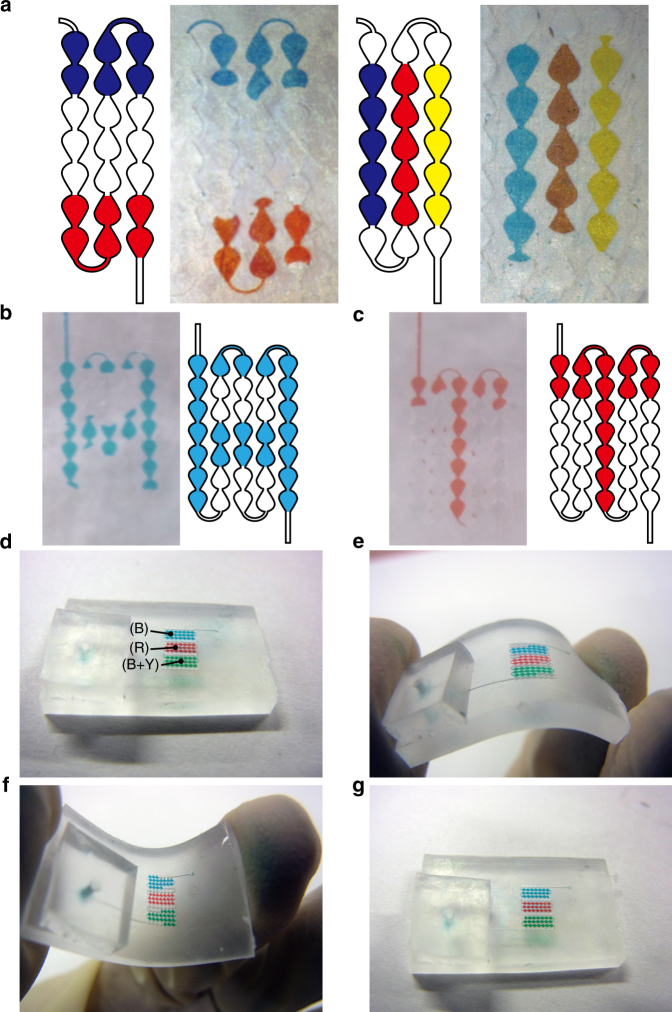
Display demonstrations. **a** Multicolored stripe patterns (vertical and horizontal) displayed on meandering microchannel. **b**, **c** Bitmap characters “A” and “T” on microfluidic-based reflective display. **d**–**g** Bending test of the display. The display image was maintained after the bending

We created images in meandering microchannels to test the feasibility of our concept of a flexible multicolor reflective display. Two types of colored stripes, which ran vertically (Fig. 8a, left) and horizontally (Fig. 8a, right and Supporting Movie 2) with respect to the direction of the microchannel, were successfully demonstrated over an area of 7 × 3 pixels. Examples of the characters A and T were displayed as bitmap graphics in a region of 7 × 5 pixels (Fig. 8b, c). These images were maintained in a stable state by stopping the suction system with no energy supply and were not disturbed by handling or by the orientation of the display with respect to gravity. These results show that our system can display multicolored reflective images and retain them with no energy consumption.

The durability of the displayed images under bending was also examined. A three-color striped image was created in an area of 11 × 7 pixels, and the display was bent to produce both concave and convex curvatures (Fig. 8d–g and Supporting Movie 3). Although the original striped image was slightly disturbed when the display was bent (Fig. 8e, f), the image was recovered when the display was returned to the initial flat condition (Fig. 8g). This demonstration indicates that our display is bendable and can maintain images when attached to flexible objects.

## Discussion

We successfully demonstrated the formation of multicolor reflective images using our proposed microfluidic-based display. Fluid-based systems, such as a camouflage skin for soft robots^18^ and electrowetting-driven droplet displays^14^, have previously been demonstrated to be reflective multicolored displays, but their functions are limited by their working principles. For instance, for camouflage skin^18^, a microfluidic channel and fluid colored by pigment are used to change the color of specific patterns on the skin surface of soft robots. However, the design of the displayed image is defined by the patterns of the microchannel, and arbitrary colorful images cannot be displayed. In the electrowetting-driven display, arbitrary multicolor bitmap images can be shown, but an electric power supply is needed to control the surface hydrophilicity and retain the image. By contrast, because of the design of the microchannels and precise fluid control, our proposed system can display arbitrarily colored bitmap images using dyed droplets and maintain a stable image with no energy consumption.

For our display system, the microchannel dimensions are important parameters for determining its performance. The pixel resolution of the display described in this paper is ~50 dpi, which is relatively low compared to commercially available reflective displays (Kindle Paperwhite, Amazon: 350 dpi; reMarkable paper tablet, reMarkable: 226 dpi; DPT-RP1, Sony: 206 dpi). Using our approach, it is possible to downsize the microchannel design to achieve a higher-resolution display: a microchannel with an ~100 μm dot pixel diameter and ~10 μm line width, corresponding to ~200 dpi, could be fabricated by the same soft lithography processes.

In addition to the pixel resolution, the minimum differential pressure *P*_min_ is affected by the length and cross-sectional area of the microchannels. The typical length and cross-sectional area of the microchannels presented here is ~30 mm and ~80,000 µm^2^, respectively. In practical use, *P*_min_ is proportional to the length of the microchannel, inversely proportional to the cross-sectional area, and proportional to the number of liquid/air interfaces. Thus, high differential pressures are required for displays with large areas or high pixel resolutions. For example, ~1 MPa would be required to control a 200 dpi display with an area of 1 cm^2^. To operate displays with large areas or high pixel resolutions, a parallel arrangement of short-length microchannels could effectively decrease the value of *P*_min_.

The number of colors that can be displayed in our system is determined by the number of dyed water types (inks) prepared in the liquid selector. Here, we demonstrated the use of three primary color pigments, suggesting that all colors can be adopted for the display by mixing these three pigments. In the system described in this paper, the color switching enabled by the liquid selector, which is attached outside the microchannel, requires manual preparation of the required colors; however, it would be possible to prepare all colors on-chip by integrating microfluidic valves and mixers^22^ into our display system.

One attractive feature of our display system is its transparency and flexibility. Flexible electronic devices^23,24^, such as flexible tactile sensor array sheets^25–27^ and flexible displays^9,28,29^, have recently received increased attention as components of wearable or attachable electronic devices, but the integration of fluidic systems into such thin and flexible devices has not been explored. The microfluidic display presented here is ~3 mm thick, but it would be possible to make a display as thin as 300–500 µm. A thin, flexible, and transparent reflective display can be attached to various curved objects or devices and can show images overlapping the surface textures below the display. We believe that our display system can open a new avenue for providing color and design changeability to objects, such as bags, shoes, and clothes.

## Electronic supplementary material

Supporting Information SI1-SI4

Supporting Movie 1

Supporting Movie 2

Supporting Movie 3
