# Supplementary material for: Microfluidic-based flexible reflective multicolor display
Source: Microsyst Nanoeng. 2018 Jul 16;4:17. doi: 10.1038/s41378-018-0018-1 (PMC6220178; doi:10.1038/s41378-018-0018-1)
Supplement: Supplementary file 1 — Supporting Information SI1-SI4 [file 41378_2018_18_MOESM1_ESM.pdf]

Supporting Information

Microfluidic-based flexible reflective multicolour display

Kazuhiro Kobayashi, *Hiroaki Onoe*

Table of Contents

SI1. Device fabrication (Figure S1-1, Figure S1-2)

SI2. Images of the whole setup (Figure S2)

SI3. Reflection spectra of dyed water (Figure S3-1, Figure S3-2)

SI4. Relationship between water loss and number of continuous dots (Figure S4)

Supporting Information SI1. Device fabrication

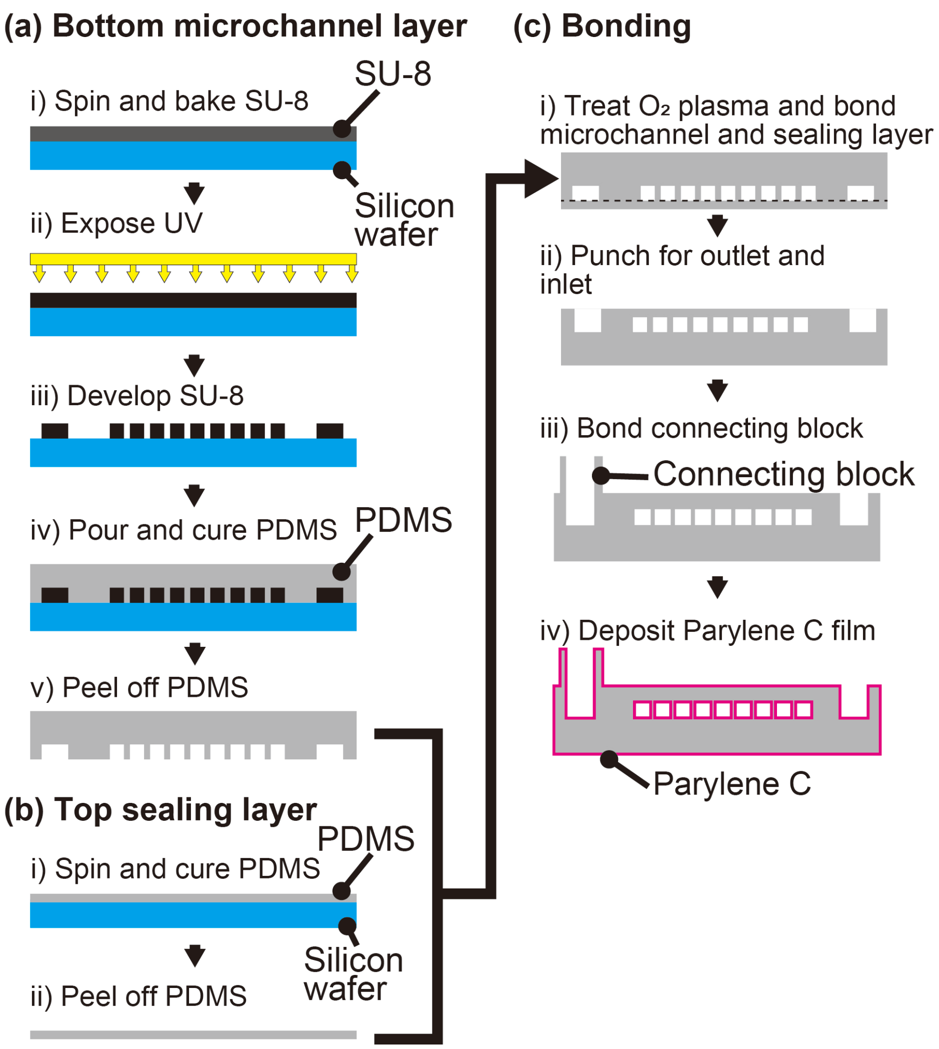

Figure S1-1. Fabrication processes of the device. **(a)** Fabrication of the bottom microchannel layer. **(b)** Fabrication of the top sealing layer. **(c)** Bonding of both layers.

The device fabrication processes were divided into three parts: The fabrication of the bottom microchannel layer (Figure S1-1 (a)), the fabrication of the top sealing layer (Figure S1-1 (b)), and bonding of both two layers (Figure S1-1 (c)).

The fabrication process of the bottom microchannel layer was as follows. Mould masters for the bottom microfluidic channels were fabricated on a silicon wafer

using standard photolithography techniques (Figure S1-1 (a)-i), (a)-ii), and (a)-iii)). A silicon wafer was coated with negative photoresist (SU-8 3050) using a spin coater at 1200 rpm for 30 s (80 μm in thickness). Next, the coated silicon wafer was placed on a hotplate at 95°C for 50 min. The silicon wafer was then coated again with the negative photoresist using a spin coater at 1200 rpm for 30 s (total 200 μm in thickness). The coated silicon wafer was placed on a hotplate at 95°C for a further 50 min (Figure S1-1 (a)-i)). A photomask with the microchannel pattern was then contacted on the photoresist layer and exposed to UV light for 15 s with a mask aligner (EMA-400, Union Optics Co., Ltd.) (Figure S1-1 (a)-ii)). Note that the photomasks were designed using Adobe Illustrator CC and photomask fabrication was outsourced to Tokyo Process Service Co. The exposed photoresist layer was then developed using an SU-8 developer for 10 min, followed by three sets of rinsing with IPA and drying by blowing with N_2 (Figure S1-1 (a)-iii)).

The PDMS prepolymer and curing agent were mixed in a 10:1 ratio and cured on the patterned SU-8 mould master, thoroughly degassed in a vacuum, and cured at 40°C for 12 h to form pixel-patterned microchannels (Figure S1-1 (a)-iv)). At the same time, the mixture was cast onto another silicon wafer with spin coating at 750 rpm for 5 min to form a PDMS sheet (~20 μm) (Figure S1-1 (b)-i)). After curing, both the PDMS microchannel and sheet were peeled off from the silicon wafers (Figure S1-1 (a)-v) and (b)-ii)). After oxygen plasma treatment (100 W for 1.5 min, SEDE-P, Meiwafosis Co., Ltd.), both the microchannel and the sealing layers were bond (Figure S1-1 (c)-i)). After bonding, the bonded PDMS microchannels were punched to make holes for an inlet and an outlet for tubing (Figure S1-1 (c)-ii)). Subsequently, a punched PDMS block (connecting block) for fixing the tubing properly was bond onto the outlet (Figure S1-1

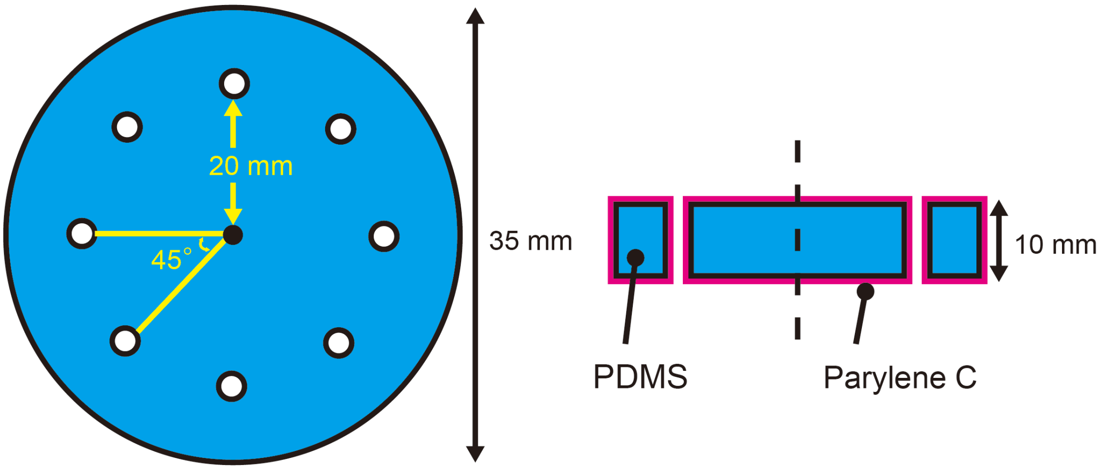

Figure S1-2. Design of the liquid selector.

(c)-iii)). Finally, an approximately 5-nm-thick Parylene C film was deposited inside the microchannel using parylene vapour deposition apparatus (PDS-2010, Japan Parylene Co.) to prevent air leakage inside the microchannel (Figure S1-1 (c)-iv)). The thickness of Parylene C film was estimated by depositing Parylene C on a sample glass plate simultaneously with the device and by measuring its thickness by a stylus profiler (Dektak, Bruker).

The liquid selector was made of PDMS (Figure S1-2). The PDMS prepolymer and curing agent were mixed in a 10:1 ratio and cured on the sterol's petri dish (NH-52, AS ONE Corp.), thoroughly degassed in a vacuum, and cured at 40°C for 12 h. The height of the liquid selector was adjusted to 10 mm. After curing, we punched six holes (φ = 1.5 mm) at 45° intervals 20 mm from the centre of the liquid selector. Finally, Parylene C film was deposited for approximately 5 nm on the surface.

Supporting Information SI2. Images of the whole setup.

Figure S2 shows images of the whole setup including the microfluidic device, liquid selector, and computer-controlled valve system.

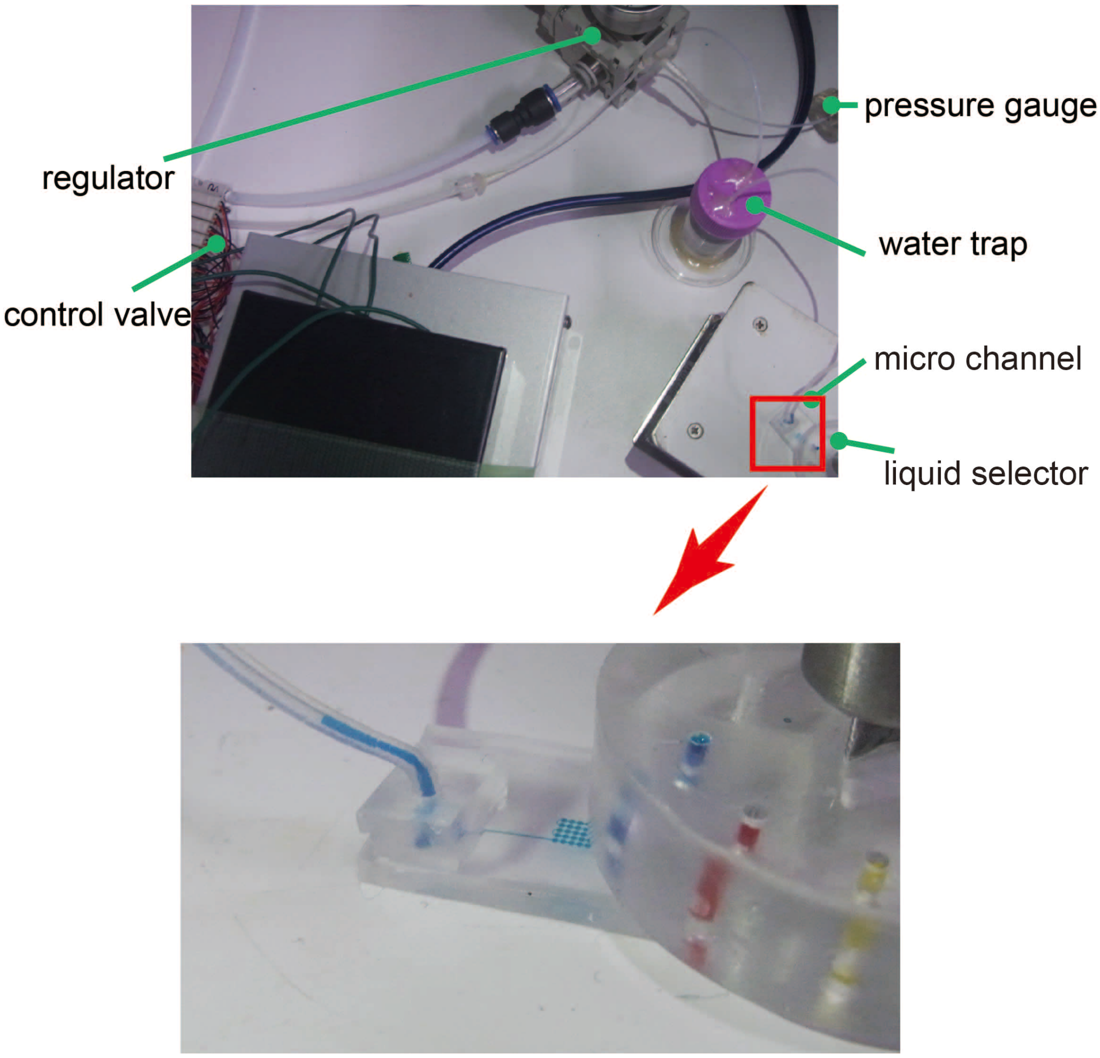

Figure S2. Images of the whole setup.

Supporting Information SI3. Reflection spectra of dyed water

Figure S3-1 shows reflection light spectra of the dyed water in Figure 7. The droplets of the dyed water in the microchannel were observed with an upright microscope (BX-50, Olympus). Their reflection light spectra were analysed by using a UV-vis NIR spectrometer (USB2000+, Ocean Optics). The corresponding light coordinates for each colour were shown in Figure S3-2.

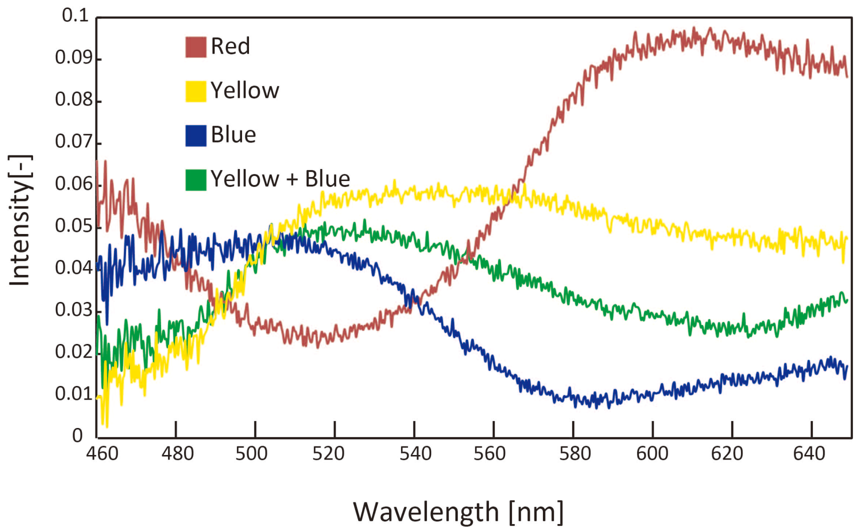

Figure S3-1. Reflection light spectra of the dyed water in the microchannel.

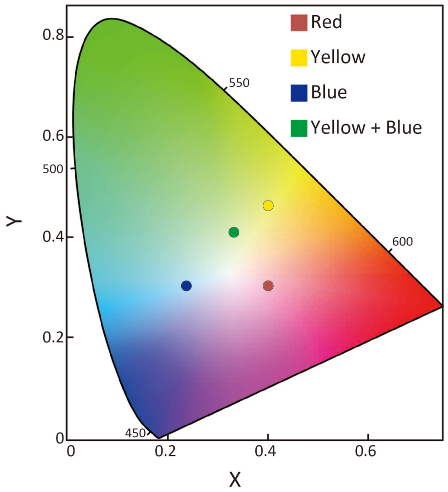

Figure S3-2. Corresponding reflection coordinates in the Commission Internationale de l'Eclairage (CIE)-1931 of each dyed water.

Supporting Information SI4. Relationship between water loss and number of continuous dots

Figure S4 shows W_{loss} for varied number of continuous dots of dyed water droplets. There was no significant difference in the values of W_{loss} between the number of continuous pixels. It indicates the water loss occurs depending on the channel geometry, not on the separated number of continuous dyed water pixels.

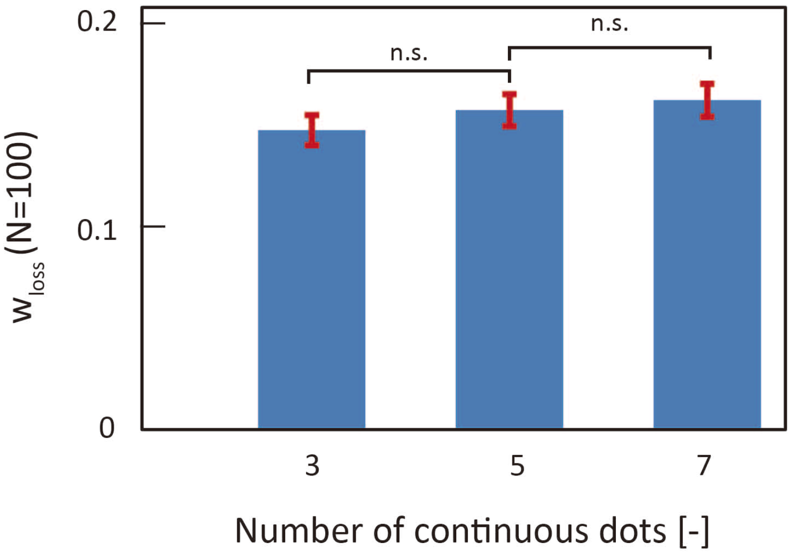

Figure S4. Relationship between W_{loss} and the number of continuous dots (number of data for each condition: n=20).
